# Cutaneous Graft-Versus-Host Disease After Allogeneic Hematopoietic Stem Cell Transplantation: A Single-Center Retrospective Cohort Study of Clinical Spectrum and Treatment Patterns

**DOI:** 10.3390/life16081255

**Published:** 2026-07-29

**Authors:** Annunziata Raimondo, Annunziata Nigro, Mara Corbisieri, Valentina Giudice, Bianca Serio, Serena Lembo, Carmine Selleri

**Affiliations:** 1 Department of Medicine Surgery and Dentistry, “Scuola Medica Salernitana”, University of Salerno, 84084 Salerno, Italy; annnigro@unisa.it (A.N.); m.corbisieri1@studenti.unisa.it (M.C.); slembo@unisa.it (S.L.); cselleri@unisa.it (C.S.); 2 Graduate School in Clinical Pathology and Clinical Biochemistry, University of Salerno, Baronissi, 84081 Salerno, Italy; 3 Hematology and Transplant Center, University Hospital “San Giovanni Di Dio E Ruggi d’Aragona”, 84131 Salerno, Italy; bianca.serio@sangiovannieruggi.it

**Keywords:** cutaneous graft-versus-host disease, allogeneic stem cell transplantation, ruxolitinib, steroid-refractory GVHD, real-world evidence

## Abstract

Cutaneous graft-versus-host disease (GVHD) is a common complication of allogeneic haematopoietic stem cell transplantation (allo-HSCT). Despite established guidelines, discrepancies persist between clinical trial evidence and real-world practice, particularly for newer agents such as ruxolitinib. To characterize the phenotypic spectrum of cutaneous GVHD, evaluate real-world treatment strategies—with a specific focus on systemic and topical ruxolitinib—and assess adherence to national and international guidelines, we conducted a retrospective observational study of patients undergoing allo-HSCT between 2015 and 2025 who were referred to a dedicated dermato-haematology clinic. Clinical, histological, and therapeutic data were collected. Cutaneous manifestations were classified according to National Institutes of Health (NIH) and Italian Group for Blood and Marrow Transplantation (GITMO) criteria. Of 62 transplanted patients, 44 (71%) developed GVHD, with the skin as the most frequently involved organ. Acute GVHD predominantly presented with maculopapular eruptions, whereas chronic GVHD showed heterogeneous phenotypes, including sclerotic variants. First-line management was largely guideline-concordant. Systemic ruxolitinib was administered to 3% of patients in the aGVHD group and 3% in the cGVHD group. Steroid-refractory and steroid-dependent status was not systematically recorded; therefore, treatment eligibility could not be reliably determined, and the observed frequencies should not be interpreted as evidence of underuse. Topical ruxolitinib was not used and remains investigational for cutaneous GVHD. Interpretation should also consider that the study period encompassed changes in regulatory approval, reimbursement, and access to targeted therapies. Structured multidisciplinary assessment may support the management of complex cutaneous GVHD, although its effects on treatment decisions and patient outcomes require prospective evaluation.

## 1. Introduction

Graft-versus-host disease (GVHD) remains a major complication of allogeneic hematopoietic stem cell transplantation (allo-HSCT) and continues to contribute substantially to transplant-related morbidity and mortality despite advances in donor matching and immunosuppressive strategies [1,2].

The skin is the most frequently involved organ in both acute and chronic GVHD and often represents the earliest manifestation of systemic alloimmune activation [3].

The biological mechanisms underlying cutaneous GVHD evolve across the trans-plant course. In aGVHD, conditioning-related tissue injury and microbial signals pro-mote antigen presentation, donor T-cell activation, cytokine amplification, and keratinocyte damage. Chronic disease involves more complex immune dysregulation, including persistent T- and B-cell activation, defective immune tolerance, macrophage-mediated inflammation, and fibroblast activation. These processes help explain the transition from inflammatory eruptions to lichenoid, poikilodermatous, or sclerotic manifestations. They also support the clinical need for repeated examination, because phenotype and severity may change over time and may require adjustment of both skin-directed and systemic treatment [1,3,4,5]. Early recognition may therefore help prevent progression to more extensive inflammation, fibrosis, and functional impairment.

Acute GVHD (aGVHD), typically occurring within 100 days after transplantation, most commonly presents with erythematous maculopapular eruptions. Chronic GVHD (cGVHD), which may develop later or overlap with acute disease, exhibits greater clinical heterogeneity and can resemble autoimmune or fibrosing dermatoses, with manifestations ranging from lichenoid lesions to sclerodermiform changes [6,7]. Accurate phenotypic classification is therefore essential for appropriate staging and treatment selection.

Cutaneous involvement is clinically relevant not only because it may be the first recognisable sign of GVHD, but also because persistent inflammation, pruritus, pain, barrier disruption, pigmentary change, ulceration, and progressive sclerosis can impair daily activities and quality of life. Dermatological assessment may nevertheless be challenging in the post-transplant setting. Drug eruptions, viral exanthems, engraftment syndrome, toxic erythema, contact dermatitis, and other inflammatory dermatoses may resemble acute or chronic GVHD. Diagnosis therefore requires integration of lesion morphology and distribution with chronology, extracutaneous findings, medication exposure, and the overall transplant course. Histopathology can support the diagnosis in atypical cases, but clinicopathological correlation remains essential because individual histological findings are not entirely specific [3,8,9].

Consensus criteria from the National Institutes of Health (NIH) and national recommendations from the Italian Group for Blood and Marrow Transplantation (GITMO) provide standardized definitions and organ-specific severity scoring systems [8,10]. High-potency topical corticosteroids are recommended for skin-limited aGVHD, whereas systemic corticosteroids remain the standard for more widespread disease. In steroid-refractory disease, ruxolitinib, a selective JAK1/2 inhibitor, has demonstrated efficacy in both acute and chronic GVHD and is incorporated into contemporary treatment algorithms [4,11].

In cGVHD, systemic corticosteroids, often combined with calcineurin inhibitors, remain first-line therapy, although prolonged exposure is associated with significant adverse effects [12,13]. Ruxolitinib has shown superiority over the best available therapy in the REACH3 trial, especially in extensive or sclerotic skin, and is now officially recommended by both GITMO and NIH for steroid-refractory cGvHD [11].

Treatment selection is influenced by the extent and severity of skin disease, concurrent organ involvement, response to corticosteroids, previous immunosuppressive exposure, comorbidities, infection risk, and access to specialist therapies. Localised manifestations may be managed with topical agents and supportive skin care, whereas progressive, extensive, or multisystem disease generally requires systemic treatment. In routine practice, therapies are frequently combined, replaced, or administered sequentially as the clinical course evolves. Consequently, real-world treatment frequencies require explicit denominators and careful distinction between the number of patients treated and the number of recorded treatment entries.

Multidisciplinary approaches are increasingly recognised as important in the assessment of cutaneous GVHD. NIH and GITMO recommendations emphasise dermatological expertise in diagnosis, staging, and ongoing management [8,10]. In this setting, our dedicated dermato-haematology clinic provides structured skin assessment, phenotypic classification, biopsy in selected diagnostically uncertain cases, and multidisciplinary treatment discussion.

This retrospective real-world study describes patients evaluated in a dedicated dermato-haematology clinic at the San Giovanni di Dio e Ruggi d’Aragona University Hospital of Salerno, Italy. The objectives were to describe the phenotypic variability of cutaneous GVHD, characterise treatment patterns, and compare the observed diagnostic and therapeutic approach with GITMO recommendations. The study was designed to describe clinical practice; it was not designed to determine whether multidisciplinary care changed staging, treatment decisions, guideline adherence, or patient outcomes.

## 2. Materials and Methods

### 2.1. Study Design and Population

This retrospective observational study included all 62 consecutive patients who underwent allo-HSCT at the Bone Marrow Transplant Unit of A.O.U. San Giovanni di Dio e Ruggi d’Aragona, Salerno, Italy, between 1 January 2015 and 31 December 2025. All recipients were systematically referred for dermatological evaluation, irrespective of the presence of clinically apparent or suspected cutaneous involvement. No patients were excluded from the source cohort. Overall, 44 patients developed GVHD and constituted the GVHD-positive analytic cohort, whereas the remaining 18 recipients were considered only for the calculation of GVHD frequency.

Data were collected from electronic medical records and transplant registries. The variables examined included patient demographics, underlying haematological malignancy, transplant characteristics, GVHD type and onset, organ involvement, cutaneous phenotype, disease severity, and treatment strategies. Cutaneous phenotypes were assigned by the dermatology team in collaboration with the transplant haematology team, based on lesion morphology, anatomical distribution, clinical course, and NIH and GITMO criteria [8,10].

Acute and chronic GVHD were evaluated separately. Acute GVHD severity was classified according to the modified Glucksberg grading system, whereas chronic cutaneous GVHD was assessed using the NIH 2014 skin score.

Skin biopsy was performed when the clinical presentation was atypical or when the differential diagnosis remained uncertain. Histopathology was considered supportive but was not required when the clinical criteria were diagnostic. Because biopsy procedures and histopathological findings were not systematically coded in the retrospective database, the exact number of biopsied patients could not be reliably determined.

The study was conducted in accordance with the Declaration of Helsinki. The retrospective collection and analysis of anonymised clinical data were reviewed by the Local Ethics Committee ASL Napoli 3 Sud, which issued a favourable opinion under approval code n. 0031852, dated 14 February 2023.

### 2.2. Statistical Analysis

Statistical analyses were descriptive and were performed using Microsoft Excel (Microsoft Corp., Redmond, WA, USA). Continuous variables were summarised as median and range, whereas categorical variables were reported as counts and percentages. No patient was excluded from the source cohort because of incomplete documentation of an individual variable. For variables with incomplete information, the analysis was based on the available clinical records. No formal comparative or inferential analyses were performed, as the study was designed to provide a descriptive overview of clinical phenotypes and treatment patterns.

## 3. Results

**Patient characteristics.** A total of 62 patients underwent allo-HSCT, of whom 44 (71%) developed GVHD and constituted the GVHD-positive analytic cohort. Unless otherwise specified, subsequent analyses refer to these 44 patients. The median age was 52 years (range 23–71). The cohort included 25 males (25/44, 57%) and 19 females (19/44, 43%). Acute myeloid leukaemia was the most common diagnosis (27/44, 61%), followed by chronic myeloid leukaemia (8/44, 18%), myelodysplastic syndrome (6/44, 14%), multiple myeloma (2/44, 5%), and acute lymphoblastic leukaemia (1/44, 2%) (Table 1).

**GVHD type and onset.** The prevalence and onset of both acute and chronic GVHD were examined in a cohort of 44 patients. The results demonstrated that 48% of patients (n = 21) exhibited signs of aGVHD, while 14% (n = 6) presented with cGVHD. Furthermore, 39% of patients (n = 17) experienced both conditions sequentially or in the form of an overlap syndrome. The onset of aGVHD occurred at an average of 26 days post-transplant (SD ±14.0; interquartile range [IQR] 17–37), while cGVHD manifested later, at an average of 215 (SD ±175.0; interquartile range [IQR] 114–267) days post-transplant (Table 2).

**Organ Involvement.** In aGVHD, skin was the most frequently involved organ, and in 63% of patients, it was the sole site of involvement. Multiorgan involvement was less common but clinically important: concurrent skin and gastrointestinal involvement was reported in 21% of patients and concurrent involvement of the skin, gastrointestinal tract, and liver was reported in 13%. Isolated skin and liver involvement occurred in just 3% of patients. These findings are consistent with traditional evidence that skin is typically the initial and most common organ targeted by aGVHD [7].

In cGVHD, skin remained the premier affected organ, either alone (39%) or as part of multisystem involvement. Both skin and liver were involved in 22% of cGVHD, while 13% of the patients had involvement of the skin and gastrointestinal tract. Another 13% had involvement of the skin, gastrointestinal tract, and liver simultaneously. Complex multisystem involvement was observed only in cGVHD and not in aGVHD. Specifically, 4% of cGVHD patients manifested one of three patterns: (1) skin, eye, gastrointestinal tract, and oral mucosa; (2) skin, eye, gastrointestinal tract, and liver; or (3) skin, eye, gastrointestinal tract, oral mucosa, and liver (Table 3).

A different pattern of anatomical distribution of the cutaneous lesions was also observed between the two forms.

In cases of aGVHD, the trunk was the most frequently affected site (34%), followed by the limbs (24%) and the face (22%). The involvement of the hands and feet (10%), neck (6%), scalp (2%), and intertriginous folds (2%) was observed to be less frequent. Conversely, cGVHD manifested a more disseminated cutaneous involvement, with the trunk, extremities, and face being affected in 25% of patients. In 9% of cases, involvement was observed in the scalp and neck regions, while in 6% of cases, involvement was observed in the hands and feet. These findings reflect a larger surface area of cutaneous involvement in cGVHD compared with aGVHD. However, the prevalence of facial and limb involvement was found to be comparable in both diseases. More extensive and variable distribution in cGVHD likely reflects the chronic course of the disease and its propensity to induce fibrotic and sclerodermiform skin changes, in accordance with clinical findings already reported in the literature (Table 4) [5,9].

**GVHD severity and skin phenotypes.** For patients diagnosed with aGVHD, 60% were classified as grade I, 24% as grade II, and 16% as grade III according to the modified Glucksberg criteria. Among patients with cGVHD, 39% had an NIH 2014 skin score of 1, 22% a score of 2, and 39% a score of 3. The cutaneous presentations of the two entities differed markedly in terms of both appearance and distribution. A major distinction was observed between the erythematous maculopapular rashes of aGVHD and the heterogeneous range of lesions displayed by cGVHD, which included eczematous, poikilodermatous, sclerodermiform, and psoriasiform patterns (Table 5).

**Therapeutic strategies.** Management of cutaneous GVHD in our unit was stepwise, following national and international guidelines, starting with topical corticosteroids and progressing to systemic and targeted treatments in refractory disease [8,10]. However, the treatment decision was often guided by drug availability and infrastructure within the institution.

Topical corticosteroids formed the cornerstone of therapy for both acute and chronic stages. Betamethasone was employed in roughly 50% of the patients with aGVHD, followed by mometasone, beclomethasone, deoxymethasone, and clobetasol, each of which were employed in 13% of patients. The results concur with the GITMO guidelines that explain the first-line usage of high-potency corticosteroids in localized cutaneous disease. In cGVHD, deoxymethasone was the treatment of choice (67%), possibly due to its increased penetrability in skin and longer half-life, which are useful for fibrotic lesions.

Systemic steroids were used for severe-to-moderate disease or in patients not responding to topical treatment. In aGVHD, 15% of patients received methylprednisolone or prednisone. In cGVHD, 18% of patients received oral prednisone, whereas a lesser number received methylprednisolone (8%) or other steroids.

Both groups received adjunctive immunosuppressive medications: cyclosporine A was administered to 37% of patients with aGVHD and 31% with cGVHD, and mycophenolate mofetil was administered to 22% and 21% of patients, respectively. These medications are typically supplemented with corticosteroids, particularly when multi-organ disease is involved or if a steroid-sparing regimen is desired.

Systemic ruxolitinib was administered to 3% of patients in the aGVHD group and 3% of patients in the cGVHD group. Steroid-refractory and steroid-dependent status was not systematically recorded as a dedicated variable, and the available records did not consistently provide the steroid dose, treatment duration, timing of response or progression, and failure of steroid tapering required for reliable classification. Therefore, the number of eligible patients for ruxolitinib and the percentage of eligible patients who received it could not be determined retrospectively. The observed frequencies consequently cannot establish therapeutic underuse or guideline-discordant care.

Rituximab was utilized more frequently in cGVHD (6%) than in aGVHD (3%), which agrees with its approval for treatment of B-cell-mediated or sclerotic subtypes [14]. In very few cases of refractory cGVHD, the investigational anti-CD26 monoclonal antibody, Belomab, was employed.

It is interesting to note that extracorporeal photopheresis (ECP) therapy, which is both effective and well tolerated in the setting of skin-predominant GVHD, was not used here, a choice that was undoubtedly due to institutional limitations. Despite the existence of data supporting the use of PUVA treatment for localised sclerotic disease, this approach was not employed.

Overall, treatment behaviour was mainly guideline-based practice in early disease. Yet very low rates of use of second-line and new therapies reflect the gap between clinical evidence and practice. To address this disparity, there is a necessity for increased accessibility, interdisciplinary collaboration, and a greater emphasis on incorporating dermatological expertise into the treatment of GVHD.

## 4. Discussion

This real-world study describes the frequency and clinical variability of cutaneous GVHD and confirms the skin as a commonly involved organ in both acute and chronic disease. The dedicated dermato-haematology clinic provided structured dermatological assessment, phenotypic classification, biopsy in selected diagnostically uncertain cases, and joint therapeutic discussion. However, the study design does not allow changes in staging, management, guideline adherence, or outcomes to be causally attributed to the integrated care model.

The distribution of severity provides additional context for the recorded management patterns. Most aGVHD cases were grade I or II (84%), supporting the frequent use of skin-directed treatment and conventional first-line immunosuppression. Chronic disease showed a different profile: NIH skin score 3 was documented in 39% of patients, whereas poikilodermatous or sclerodermiform phenotypes together accounted for 35% of cases. These manifestations are clinically important because fibrotic progression may lead to restricted mobility and irreversible functional impairment. The greater proportion of multisystem involvement in the chronic group also illustrates why cutaneous findings should be interpreted within a comprehensive organ assessment rather than as an isolated dermatological diagnosis [5,6,8,9].

Recorded first-line management was broadly consistent with guideline recommendations. High-potency topical corticosteroids predominated in skin-limited acute disease, whereas systemic corticosteroids and adjunctive immunosuppressants were recorded in more extensive or multisystem presentations [10]. Chronic GVHD showed greater phenotypic and therapeutic heterogeneity, with topical corticosteroids used for localised disease and systemic corticosteroids plus immunosuppressants forming the principal systemic approach.

Systemic ruxolitinib was used in 3% of patients with aGVHD and 3% of patients with cGVHD. However, steroid-refractory or steroid-dependent status could not be reliably reconstructed from the retrospective records; consequently, eligibility for second-line ruxolitinib treatment could not be determined. These frequencies therefore should not be regarded as evidence of underuse and should be interpreted descriptively.

The 2015–2025 inclusion period represents an additional limitation because GVHD management evolved substantially during this decade, particularly with the progressive availability of ruxolitinib and its incorporation into treatment recommendations. Patients treated during the earlier years may have had more limited access to this therapy than those treated later. Consequently, the pooled frequencies may underestimate its adoption in more recent clinical practice. Because of the limited sample size, treatment patterns could not be reliably analysed according to calendar period.

The biological rationale for JAK1/2 inhibition is supported by the role of JAK/STAT signalling in cytokine-mediated T-cell activation and tissue inflammation. Clinical trials have demonstrated the efficacy of systemic ruxolitinib in steroid-refractory aGVHD and cGVHD [11,15,16,17,18,19]. These published trial outcomes should not be interpreted as outcomes of the present cohort. Cytopenias, infectious risk, haematological monitoring requirements, and local access may also influence treatment selection [20].

Topical ruxolitinib was not used in this cohort. Although early reports and phase II data suggest potential benefit for selected localised cutaneous manifestations [21,22,23,24], its role in GVHD remains investigational and it is not included in the referenced NIH or GITMO treatment recommendations.

The integrated clinic supported structured skin assessment, multidisciplinary phenotype assignment, biopsy in selected uncertain cases, and joint treatment discussion. However, pre-dermatology staging and treatment plans were not systematically recorded, and no comparator pathway without dermatology involvement was available. The study therefore cannot quantify whether dermatologist involvement changed staging or treatment decisions, accelerated recognition of sclerotic variants, improved guideline adherence, or improved patient outcomes.

Systematic referral of all allo-HSCT recipients represents an important feature of the clinical pathway, as dermatological evaluation was not restricted to patients with visible or suspected skin involvement. This approach may facilitate the recognition of subtle or evolving manifestations and provides a structured setting for documenting lesion morphology and distribution. The present study was designed to describe the organisation of the integrated service, cutaneous GVHD phenotypes, and recorded management patterns; it was not designed to quantify the specific effects of dermatological involvement on staging, treatment decisions, guideline adherence, or patient outcomes. These aspects should be evaluated in future prospective studies.

Overall survival, relapse, cause-specific mortality, quality of life, formal treatment response, corticosteroid tapering, steroid-sparing effects, and their associations with cutaneous phenotype or severity were not predefined study outcomes and were not systematically collected. Therefore, no formal analyses of these outcomes were performed. Prospective studies with standardised follow-up and predefined clinical endpoints will be required to investigate these associations.

Additional limitations include the retrospective design, single-centre setting, relatively small sample size, incomplete retrospective documentation of biopsy and histopathological data. These features limit the generalisability of the findings and the possibility of statistical inference. Prospective studies with predefined treatment eligibility criteria, standardised follow-up, and clinically relevant outcomes are required to evaluate multidisciplinary care and evolving therapeutic strategies.

Future prospective studies should enroll consecutive allo-HSCT recipients using a standardised dermatological assessment schedule and predefined definitions of steroid-refractory and steroid-dependent disease. Recording serial skin scores, biopsy findings, systemic organ staging, treatment exposure and response, corticosteroid dose, patient-reported symptoms, quality of life, relapse, survival, and mortality would permit more informative analyses. A calendar-era approach would also distinguish changes in practice associated with regulatory approval, reimbursement, and local availability of new therapies. Finally, documenting staging and treatment plans before and after multidisciplinary review would allow the contribution of dermatological input to be evaluated directly.

## 5. Conclusions

This study provides a descriptive account of cutaneous GVHD phenotypes and treatment patterns in a dedicated dermato-haematology setting. Recorded first-line therapies were broadly consistent with guideline recommendations, whereas targeted and procedural therapies were infrequently represented among treatment entries. Because eligibility for second-line therapy was not systematically documented and the 2015–2025 period encompassed substantial changes in therapeutic availability, the findings should not be interpreted as proof of current treatment underuse. Topical ruxolitinib remains investigational for GVHD and was not used in this cohort. Integrated dermatology–haematology services may support the assessment and management of complex cutaneous GVHD, but their effects on staging, treatment decisions, guideline adherence, and patient outcomes require prospective evaluation.

## Figures and Tables

**Table 1:** Demographic and clinical characteristics of the study population (*n* = 44) GVHD following HSCT.

Characteristic	Value
Median age at HSCT, years (range)	52 (23–71)
Sex, *n* (%)	Male 25 (57%) Female 19 (43%)
Primary hematological diagnosis *n* (%)	LLA 1 (2%) LMA 27 (61%) LMC 8 (18%) MDS 6 (14%) MM 2 (5%)

Abbreviations: HSCT—hematopoietic stem cell transplantation; LLA—acute lymphoblastic leukemia; LMA—acute myeloid leukemia; LMC—chronic myeloid leukemia; MDS—myelodysplastic syndrome; MM—multiple myeloma.

**Table 2:** Distribution and time to onset of acute and chronic GVHD (*n* = 44).

GVHD Type	Frequency (%)	Mean Time to Onset (Days ± SD)
aGVHD	48	26 ± 14
cGVHD	14	215 ± 175
Both (aGVHD + cGVHD)	39	-

Abbreviations: aGVHD—acute GVHD; cGVHD—chronic GVHD.

**Table 3:** Organ involvement patterns in aGVHD and cGVHD.

Organ Involvement	aGVHD (%)	cGVHD (%)
Skin	63	39
Skin + GI	21	13
Skin + liver	3	22
Skin + GI + liver	13	13
Skin + eyes + GI + mouth	–	4
Skin + eyes + GI + liver	–	4
Skin + eyes + GI + mouth + liver	–	4

Abbreviations: GI—gastrointestinal.

**Table 4:** Anatomical distribution of cutaneous lesions in aGVHD and cGVHD.

Anatomical Distribution of Cutaneous Lesions	aGVHD (%)	cGVHD (%)
Face	22	25
Neck	6	9
Scalp	2	9
Limbs	24	25
Trunk	34	25
Hands and feet	10	6
Skin folds	2	0

**Table 5:** Cutaneous manifestations observed in aGVHD and cGVHD.

Lesion Type	aGVHD (%)	cGVHD (%)
Erythematous	82	39
Eczematous	18	22
Poikilodermiform	-	17
Sclerodermiform	-	17
Psoriasiform	-	5

## Data Availability

The data presented in this study are available on reasonable request from the corresponding author. The data are not publicly available due to privacy and ethical restrictions.

## Abbreviations

The following abbreviations are used in this manuscript:

GVHD	Graft-versus-host disease
Allo-HSCT	Allogeneic hematopoietic stem cell transplantation
NIH	National Institutes of Health
GITMO	Italian Group for Bone Marrow Transplantation
aGVHD	Acute graft-versus-host disease
cGVHD	Chronic graft-versus-host disease
SD	Standard deviation
IQR	Interquartile range
ALL	Acute lymphoblastic leukemia
AML	Acute myeloid leukemia
CML	Chronic myeloid leukemia
MDS	Myelodysplastic syndrome
MM	Multiple myeloma
GI	Gastrointestinal
